# Effect of In Vitro Digestion on Bioactive Peptides Related to Immune and Gut Health in Intact Cow’s Milk and Hydrolyzed Protein-Based Infant Formulas

**DOI:** 10.3390/nu16193268

**Published:** 2024-09-27

**Authors:** Gabriela Grigorean, Xiaogu Du, Russell Kuhfeld, Elisabeth M. Haberl, Bo Lönnerdal

**Affiliations:** 1 Proteomics Core Facility, University of California, Davis, CA 95616, USA; ggrigorean@ucdavis.edu; 2 Department of Nutrition, University of California, Davis, CA 95616, USA; xdu@ucdavis.edu; 3 Nutrition Program, School of Nutrition and Public Health, College of Health, Oregon State University, Corvallis, OR 97331, USA; kuhfeldr@oregonstate.edu; 4 HiPP GmbH & Co. Vertrieb KG, 85276 Pfaffenhofen, Germany; elisabeth.haberl@hipp.de

**Keywords:** bioactive peptides, infant formula, extensively hydrolyzed infant formulas (eHFs), intact protein formulas (iPFs), immune health, gut health, prebiotics, synbiotics

## Abstract

**Background/Objectives**: Human milk is the optimal source of nutrition and protection against infection for infants. If breastfeeding is not possible, standard and hydrolyzed infant formulas (IF) are an alternative. Extensively hydrolyzed IFs (eHFs) contain bioactive peptides, but their activities have rarely been evaluated. The aim of this study was to characterize and compare the bioactive peptide profiles of different eHFs and standard IFs before and after in vitro digestion. **Methods**: Two forms, liquid and powder, of intact protein formula (iPF) and eHF were subjected to in vitro gastrointestinal digestion, mimicking a young infant’s gut (age 0–4 months) and an older infant’s gut (>6 months). Bioactive peptides of in vitro digested and undigested formulas were analysed with Liquid Chromatography–Mass Spectrometry (LC–MS). **Results**: In all samples, a variety of peptides with potential bioactive properties were found. Immuno-regulatory peptides, followed by antimicrobial and antioxidative peptides were most frequent, as were peptides promoting wound healing, increasing mucin secretion, regulating cholesterol metabolism, and preventing bacterial infection. Peptides typically found in yoghurt and colostrum were identified in some formula samples. **Conclusions**: The high amounts of bioactive peptides with various properties in eHFs and iPFs indicate a possible contribution to infection protection, healthy gut microbiomes, and immunological development of infants. eHFs showed similar compositions of bioactive peptides to iPFs, with intermittently increased peptide variety and quantity.

## 1. Introduction

Human milk is the optimal source of nutrition for infants. It contributes protective effects against infection [1] and promotes immunological development as well as a healthy gut microbiome [2,3]. Among several components, human milk provides a large variety of bioactive peptides derived from major human milk proteins such as caseins, whey proteins, and mucins [4]. Caseins and whey proteins (e.g., α-lactalbumin and lactoferrin) constitute the largest part, whereas mucins constitute only a small percentage of total protein [5]. Small amounts of bioactive peptides may already be present in human milk itself due to endogenous proteases [6]. Larger quantities of bioactive peptides are released during digestion in an infant’s stomach and small intestine [4,7]. This has been investigated in various in vitro digestion systems [8,9] and in animal models [10]. These peptides can be absorbed, can exert bioactivities, and can furthermore be found in the stool of breast-fed infants [11]. Bioactive peptides play an important role in the maintenance of the gut epithelial barrier, especially together with secretory IgA (SIgA)—an antibody found in human milk that has been shown to exert an important gut mucosa shielding effect [12].

If breastfeeding is not possible, besides infant formulas (IFs) with intact proteins, hydrolyzed formulas are also an alternative for infant nutrition [13]. Bioactive peptides in infant formulas are likewise formed from bovine milk proteins in cow’s milk formula [8], and hydrolyzed formulas originally contain a larger proportion of peptides as they have been exposed to proteases during the manufacturing process. Following gastrointestinal digestion, a variety of small peptides are detected, some of which are also biologically active. It has previously been shown that in vitro digestion of an extensively hydrolyzed IF (eHF) resulted in fewer bioactive peptides as compared to a standard IF. However, several other bioactive peptides, such as fragments of β-lactoglobulin and residues 60–70 of β-casein, were preferentially released by a combination of industrial hydrolysis and in vitro digestion [8]. Thus, standard IFs and eHFs can be expected to result in the formation of different peptides with varying bioactivities.

There have been several clinical studies on eHFs; however, besides the findings of those focusing on the treatment or prevention of allergies, information on the characteristics and properties of various types of eHFs is limited.

Interestingly, eHFs contain a variety of bioactive peptides, but outcomes based on the activities of such peptides have rarely been evaluated. Further, the quantity of bioactive peptides in IFs is essential to know in order to elicit a measurable biological effect [14]. According to recent studies, eHFs can reduce allergic sensitization to cow’s milk [15,16,17] and the development of allergic diseases in the first two years of life [18], compared to standard IFs. Some eHFs are approved for managing cow’s milk allergy [19].

Hydrolyzed and standard infant formulas are available in liquid and powdered forms, each being exposed to varying degrees of heat treatment during the manufacturing process. Heat treatment can affect digestion and the formation of bioactive peptides [20]; therefore, one aim of this study was to investigate whether the manufacturing processes of liquid and powdered IFs might affect the formation of bioactive peptides. The physical form of an IF is not only a matter of the preference of the caregiver but will also determine whether the IF can be additionally supplemented with pre- or probiotics. Dry-blended powdered IF will allow the addition of both pre- and viable probiotics (i.e., a synbiotic combination), while this is not yet possible in a liquid product, which can only be enriched with prebiotics. Since the presence of pre- or probiotics may affect the digestion of IFs due to bacterial proteases, it is of interest to compare these two types of hydrolyzed formula based on the identical hydrolysate.

The second and most important aim of this study was to characterize and compare the bioactive peptide profiles of the two different formula types: eHF, based on extensively hydrolyzed protein, vs. standard IF, based on intact cow’s milk protein (intact protein formula (iPF)). Therefore, both the variety of bioactive peptides and the overall peptide quantity were compared. In particular, two formula types (powder and liquid) of each eHF and iPF were used. All four formulas were exposed to three in vitro digestion states: undigested and two conditions mimicking the gut of a young infant (stomach pH of 4, for 15 min) [4] and of an older infant (stomach pH of 2, for 30 min). The results were then compared to databases of cow’s milk bioactive peptides to identify peptides with known physiological activities.

## 2. Materials and Methods

### 2.1. Materials

Standard cow’s milk IF (HiPP Pre Bio COMBIOTIK^®^) and eHF (HiPP Pre HA COMBIOTIK^®^) were provided by HiPP GmbH & Co. Vertrieb KG (Pfaffenhofen, Germany). For each of the two formulas, one liquid (L) and one powdered (P) product were analyzed. The formulas were subjected to in vitro gastrointestinal digestion mimicking the physiological gastrointestinal conditions of a young infant (0–4 months of age). Additionally, comparisons to gastrointestinal conditions in older infants (>6 months of age) were performed. A description of the investigated materials is presented in Table 1.

### 2.2. Peptide Samples Preparation

Sample preparations and analyses were carried out as biological duplicates. In vitro digestion was performed according to our previously published work [4]. Formulas were acidified to pH 4 (young infant stomach pH) or pH 2 (older infant stomach pH) with 1 mM HCl. Pepsin (Sigma, St. Louis, MO, USA) was added as 2% pepsin in 1 mM HCl in PBS, at a ratio of 22 µL per 25 mL of sample, to achieve a protein/pepsin ratio of 12.5:1. Pepsin activity was 56 U/mL of sample. After pepsin addition, samples were incubated at 37 °C for 15 min (young infant) and 30 min (older infant). Following incubation for 15 and 30 min with pepsin, pH was adjusted back to 7.0 using NaHCO_3_, after which pancreatin (Sigma, St. Louis, MO, USA) as a 0.4% solution in 1 mM NaHCO_3_ was added to the samples at a ratio of 4 µL pancreatin per 25 mL of formula, to achieve a protein:pancreatin ratio of 62.5:1. After pancreatin addition, samples were incubated for 15 and 30 min, respectively, and enzymes were then inactivated by placing the samples in a water bath at 85 °C for one min.

Peptidomic analysis of the undigested and digested formulas was performed by the Proteomics Core Facility (UC Davis, CA, USA). Undigested eHF and iPF, and the digested formulas were spun on 10 kDa filters (Millipore, Burlington, MA, USA) for 5 min to separate peptides from proteins larger than 10 kDa, obtaining two fractions: one for peptidomic analysis and another for proteomics analysis. The proteins fraction and any peptides above 10 kDa were subjected to trypsinization in order to obtain peptide sizes that were compatible with typical Liquid Chromatography–Mass Spectrometry (LC–MS) analyses. Both the gastrointestinal digestion under 10 kDa peptides and the above 10 kDa trypsinized peptides were then desalted and subjected to LC–MS.

### 2.3. Liquid Chromatography–Mass Spectrometry (LC–MS) Analysis

Nano-liquid chromatography (nLC) separation was performed on an Easy-nLC II High-Pressure Liquid Chromatography system (HPLC) (Thermo Fisher Scientific, Waltham, MA, USA). The trypsinized peptides were reconstituted in 2% acetonitrile/0.1% trifluoroacetic acid and 1 µg of each sample was loaded onto the LC–MS system. For the LC part, samples were first sent onto a 100 µm × 25 mm Magic C18, 100 Å, 5 µm reverse phase trap where they were desalted on-line, then directly eluted onto an analytical column for on-line separation. The analytical column is a 75 µm × 150 mm Magic C18, 200 Å, 3 µm reverse phase column. Peptides were then eluted into the mass spectrometer directly from this analytical column using a 2-buffer gradient: buffer A being 0.1% formic acid in water, and buffer B 0.1% formic acid in 100% acetonitrile, at a flow rate of 300 nL/min. The 95 min LC gradient is as follows: 0–80 min goes from 5% to 40% acetonitrile; 80–85 min goes from 35% to 80% acetonitrile; stays at 80% acetonitrile for 3 min; then goes from 80% to 5% acetonitrile over 2 min; and finally held at 5% acetonitrile for 5 min.

Mass spectra were collected on an Orbitrap Q Exactive mass spectrometer (Thermo Fisher Scientific) in a data-dependent mode with one MS precursor scan, resolution 70,000, followed by 15 MS/MS scans, resolution 17,500.

### 2.4. Data Analysis

The LC–MS raw files were processed with Proteome Discoverer 2.5 (Thermo Fisher) using the integrated SEQUEST engine. Precursor mass tolerance was set to 10 ppm. Fragment ion tolerance was 0.6 Da when using the ion trap analyzer. Trypsin, pepsin, chymotrypsin, and elastase (pancreatin) were specified as proteases and a maximum of two missed cleavages was allowed. Carbamidomethylation on cysteine (+57.021 Da) was set as static modification. Dynamic modifications included oxidation on methionine (+15.995 Da), deamidation of asparagine and glutamine (0.985 Da) and phosphorylation on serine, threonine, and tyrosine (+79.966 Da). All data were searched against the Bos Taurus Uniprot UP0009136 reference proteome without isoforms (37,511 entries), and a database of 112 common laboratory contaminants [21]. A false discovery rate of 1% was set at the PSM level as well as at 1% at the protein level. We accepted identifications with at least one unique peptide and XCorr scores of above 2 for doubly charged and of above 3 for triply charged tryptic peptides. The ptmRS node was used to localize phosphorylation sites, with a probability of 90% or higher considered to be a confident indicator of a phosphorylation site.

For label-free relative quantitation, peptides contributing to the protein abundance values were defined as those with over four points under the curve. This ensured a robust quantitation value.

Bioactive peptides were identified by manually examining the Proteome Discoverer 2.5 (Thermo Fisher)-generated peptides list. To expand, identification was performed against the appropriate databases (according to the provenience of milk samples), plus an equal number of reverse sequences and 60 common laboratory contaminant proteins, assuming a non-specific digestion enzyme. Peptides derived from major bovine milk proteins of interest were submitted to the Milk Bioactive Peptide Database [6,22,23]. This database classifies milk peptides based on biological functions that the database creators have gathered from over 260 primary research articles on milk from several mammalian species, such as cows, humans, camels, goats, and more. Several other milk databases were also searched for any peptides not found using the literature search. The search results were further corroborated by browsing reference articles.

## 3. Results

**Figure 1:**
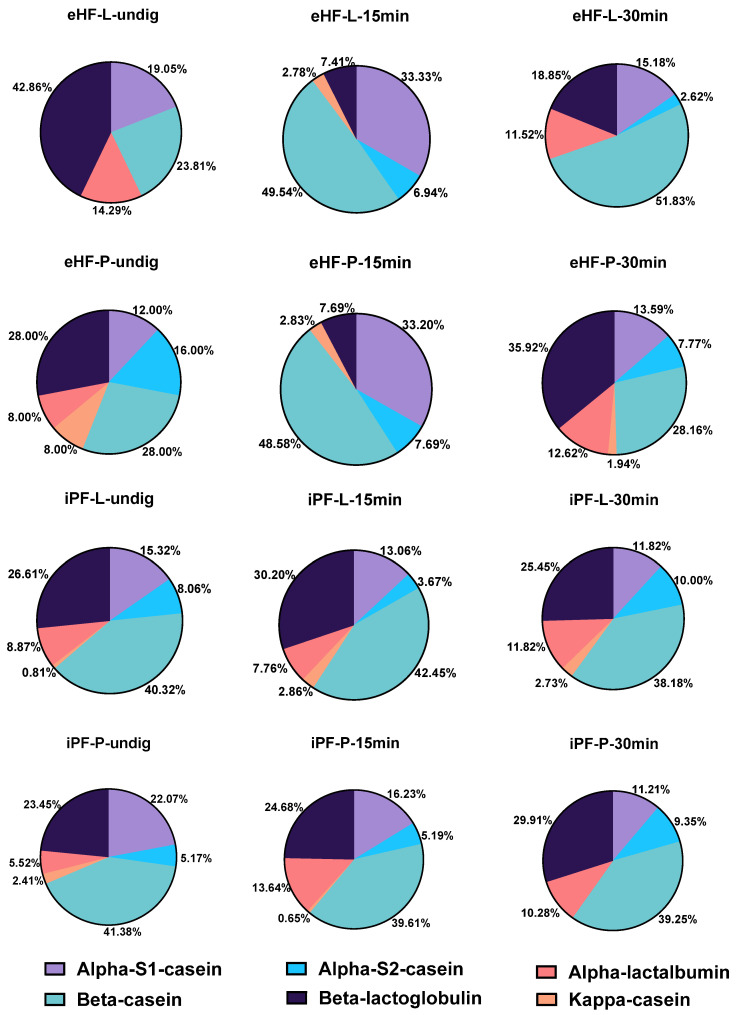
Percentage of milk protein-derived peptides found in the 12 formula samples.

Different bioactive peptides derived from major bovine milk proteins were found in the 12 formula samples during in vitro gastrointestinal digestion, including caseins and whey proteins (such as β-lactoglobulin, and lactoferrin). In all 12 formulas, casein peptides were the most commonly found peptides, specifically from α-S1-casein, β-casein, α-S2-casein, and κ-casein (overall > 97% of all peptides). Among these, β-casein derived peptides were the most common peptides (see Figure 1).

Overall, the most commonly found bioactive peptides by their associated biological functions across all samples were immuno-regulatory peptides, as well as peptides with antimicrobial, antioxidative, wound healing, increasing mucin secretion, cholesterol regulation, and *Salmonella* antivirulence activities. Additionally, peptides known from yoghurt and colostrum were found. An overview of the most commonly found bioactive peptides in formula samples after 15 min of in vitro digestion is presented in Table 2.

**Figure 2:**
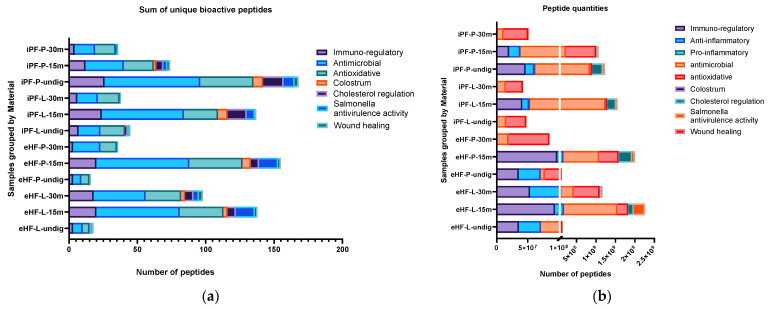
(**a**) Sum of unique bioactive peptides per peptide group found in the 12 formula samples. (**b**) Number of bioactive peptides per peptide group found in the 12 formula samples.

The quantity of bioactive peptides of the nine most relevant peptide groups (as per their associated function or source), as well as the sum of unique bioactive peptides were analyzed per sample and are depicted in Figure 2. For the peptide quantities, the integral of the respective peptide curve was calculated (i.e., the area under the curve), which represents the number of peptides present in a sample. Since this value is a unitless value, in order to present and discuss the peptide quantity values, the description ‘quant value’ will be used in the following sections.

The largest sum of unique bioactive peptides was found in the undigested iPF-P sample (170 unique bioactive peptides), followed by the samples of eHF-P (156), iPF-L (149), and eHF-L (143), all after 15 min of in vitro digestion (Figure 2a). Antimicrobial and antioxidative peptides were the most common bioactive peptides found, with antimicrobial peptides having the highest overall absolute quantity of peptides (antimicrobial: total of >4.8 × 10^9^, quant value; antioxidative: total of >2.5 × 10^9^ quant value) and antioxidative peptides being the most common peptide group in eight of 12 samples (Figure 2b). Other peptides found with high quantities were cholesterol metabolism regulation (>1 × 10^9^ quant value), *Salmonella* antivirulence activity (>477 × 10^6^ quant value), immuno-regulatory (including osteopontin and peptides with functions related to T cell response) (>426 × 10^6^ quant value), anti-inflammatory (>318 × 10^6^ quant value), and wound healing (>66 × 10^6^ quant value).

Furthermore, peptides known from colostrum were found, albeit in lower amounts, in six of the 12 samples (>39 × 10^6^ quant value). Small amounts of pro-inflammatory peptides were found in eight of the 12 samples (>6 × 10^6^ quant value).

The following sections focus on the analysis of each peptide group across all formula samples.

### 3.1. Immuno-Regulatory Peptides

#### 3.1.1. Overview of Immuno-Regulatory Peptides

**Figure 3:**
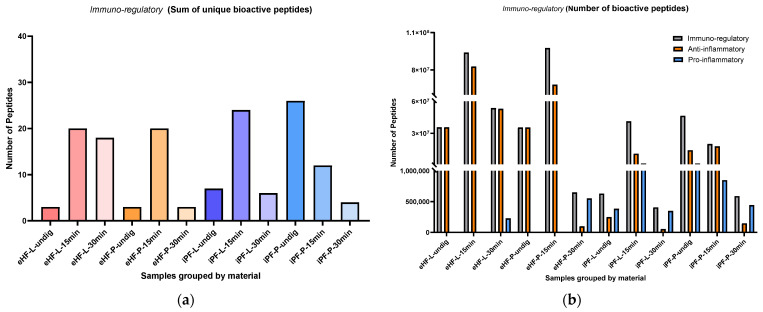
(**a**) Sum of unique bioactive peptides found among immuno-regulatory peptides per formula sample and digestion status; (**b**) Number of bioactive peptides found among immuno-regulatory peptides per formula sample and digestion status.

The sums of unique immuno-regulatory peptides (i.e., variety of peptides) per sample and digestion status are presented in Figure 3a. The highest sum of unique immuno-regulatory peptides was identified in the undigested iPF-P sample (26 unique immuno-regulatory peptides), followed by iPF-L after 15 min of in vitro digestion (24 peptides), eHF-L and eHF-P, each after 15 min of in vitro digestion (each 20 peptides), and eHF-L after 30 min of in vitro digestion (18 peptides).

Across all the samples, the highest sums of unique immuno-regulatory peptides were found in samples after 15 min of in vitro digestion; except for iPF-P, where the highest sum was found in the undigested state. In contrast, the lowest sums of unique immuno-regulatory peptides were found in samples after 30 min of in vitro digestion; except for eHF-L, where the undigested state showed the lowest variety of peptides.

Overall, there were no distinct differences between the powder and liquid samples for both formula types (eHFs and iPFs) except for the eHF samples after 30 min of in vitro digestion, where eHF-L showed a higher variety of peptides.

The numbers of immuno-regulatory peptides (i.e., quantity) per sample and digestion status are presented in Figure 3b. The highest quantities of immuno-regulatory peptides were found in the eHF-P sample (>97 × 10^6^ quant value of immuno-regulatory peptides) and the eHF-L sample (>93 × 10^6^ quant value), each after 15 min of in vitro digestion, followed by the eHF-L sample after 30 min of in vitro digestion (>53 × 10^6^ quant value). Across the iPFs, the highest quantities of immuno-regulatory peptides were found in the undigested iPF-P sample (>46 × 10^6^ quant value) and in the iPF-L sample after 15 min of in vitro digestion (>41 × 10^6^ quant value).

For the anti-inflammatory peptides, the highest quantity was found in the eHF-L sample (>82 × 10^6^ peptides), followed by eHF-P sample (>68 × 10^6^ peptides), each after 15 min of in vitro digestion, and in the eHF-L sample after 30 min of in vitro digestion (>53 × 10^6^ quant value). Across the iPFs, the highest quantities of anti-inflammatory peptides were found in the iPF-P sample after 15 min of in vitro digestion (>17 × 10^6^ quant value) and in the undigested iPF-P sample (>14 × 10^6^ quant value).

Pro-inflammatory peptides were found in all iPF samples, with the highest quantities in undigested iPF-P and iPF-L after 15 min of in vitro digestion (each >1 × 10^6^ quant value). In the eHFs, pro-inflammatory peptides were only found after 30 min of in vitro digestion for both powder and liquid samples.

After 15 min of in vitro digestion, the quantities of immuno-regulatory and anti-inflammatory peptides were at their highest for all samples; except for iPF-P, where the immuno-regulatory peptides were higher in its undigested state. Pro-inflammatory peptides were not detected in eHFs after 15 min of in vitro digestion.

Overall, there were no pronounced differences between the powder and liquid samples for both formula types (eHFs and iPFs) except for eHF samples after 30 min of in vitro digestion, where eHF-L showed a higher quantity of peptides.

Additionally, some immuno-regulatory peptides related to allergic diseases or allergic sensitization were found. The peptide Asn-Pro-Trp-Asp-Gln (NPWDQ, amino acids 107–111 of αs2-casein), which is known to inhibit intestinal allergen permeation [24], was found in several samples of iPF. It was found in all digestion statuses of the iPF-L formula and in the undigested iPF-P formula.

Further, the peptide LAYFYPE (amino acids 142–149 of αs1-casein), which is known to secrete IFN-γ, a potent inhibitor of Th2-dependent events, including IgE production [25], was found in two samples of iPF. It was found in the undigested iPF-P formula and in the iPF-L formula after 15 min of in vitro digestion.

#### 3.1.2. Lactoferrin

**Figure 4:**
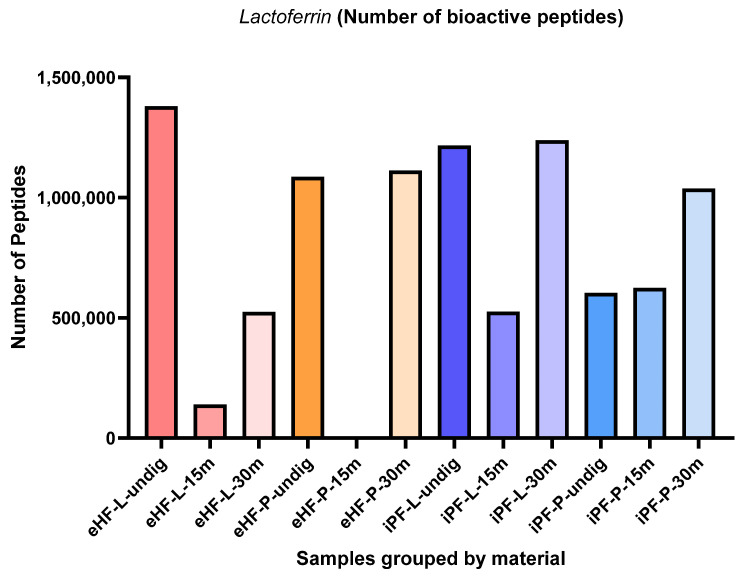
Number of lactoferrin peptides per formula sample and digestion status.

The numbers of lactoferrin peptides per sample and digestion status are presented in Figure 4. The highest quantity of lactoferrin was found in the undigested eHF-L sample (>1.3 × 10^6^ quant value for lactoferrin peptides), followed by iPF-L (>1.2 × 10^6^ quant value) after 30 min of in vitro digestion, undigested iPF-L (>1.2 × 10^6^ quant value), eHF-P (>1.1 × 10^6^ quant value) after 30 min of in vitro digestion, and undigested eHF-P (>1 × 10^6^ quant value). No lactoferrin peptides were found in the eHF-P sample after 15 min of in vitro digestion.

Across all samples, the highest quantities of lactoferrin peptides were found in samples after 30 min of in vitro digestion; except for eHF-L, where the highest quantity was found in the undigested state.

After 15 min of in vitro digestion, the quantity of lactoferrin peptides was lowest for all samples; except iPF-P, where the lactoferrin peptide quantity was slightly lower in its undigested state.

Overall, the quantities of lactoferrin peptides in the powder and liquid samples of both formula types were somewhat similar. In the eHF samples, eHF-L showed a higher quantity of lactoferrin peptides in the undigested state and after 15 min of in vitro digestion, although eHF-P dominated in quantity after 30 min of in vitro digestion. In the iPF samples, iPF-L also showed a higher quantity of lactoferrin peptides in the undigested state and after 30 min of in vitro digestion, although iPF-P was higher after 15 min of in vitro digestion.

#### 3.1.3. T Cell Response

Across all samples, two bioactive peptides with functions related to T cell response were found: polymeric immunoglobulin receptor (pIgR) [26] and glycosylation-dependent cell adhesion molecule 1 (GlyCAM1) [27] peptides. PIgR is a 100–120 kDa type 1 transmembrane protein and is involved in the transport of IgM and IgA in the gut epithelium. GlyCAM1 is a mucin-like endothelial glycoprotein and is part of the milk mucin complex.

**Figure 5:**
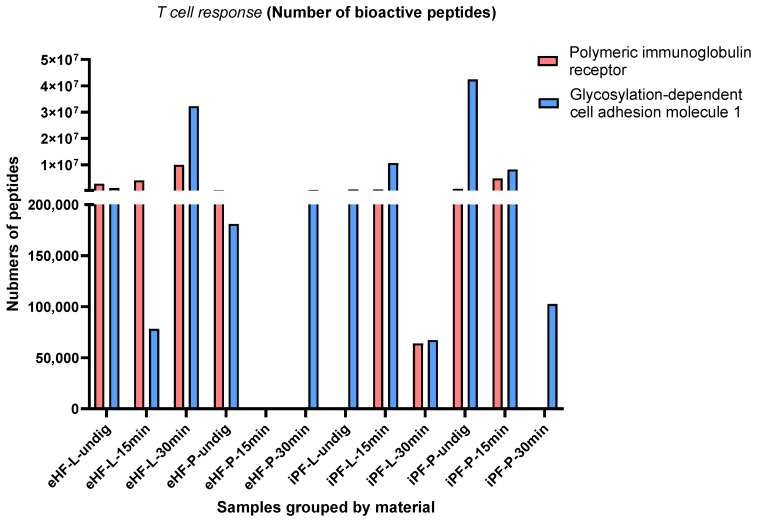
Number of the two peptides with functions related to T cells’ polymeric immunoglobulin receptor (pIgR) and glycosylation-dependent cell adhesion molecule 1 (GlyCAM1) per formula sample and digestion status.

The numbers of pIgR and GlyCAM1 peptides per sample and digestion status are presented in Figure 5. The highest quantity of pIgR peptides was found in the eHF-L sample (>9.9 × 10^6^ pIgR peptides quant value) after 30 min of in vitro digestion, followed by iPF-P (>4.8 × 10^6^ quant value) and eHF-L (>4.0 × 10^6^ quant value), each after 15 min of in vitro digestion, and undigested eHF-L (>2.8 × 10^6^ quant value).

The highest quantity of GlyCAM1 peptides was found in the undigested iPF-P sample (>42 × 10^6^ quant value for GlyCAM1 peptides), followed by eHF-L (>32 × 10^6^ quant value) after 30 min of in vitro digestion, and iPF-L (>10 × 10^6^ quant value) and iPF-P (>8 × 10^6^ quant value), each after 15 min of in vitro digestion.

No pIgR peptides were found in undigested iPF-L, iPF-P, and eHF-P after 30 min of in vitro digestion. Neither pIgR nor GlyCAM1 peptides were found in the eHF-P formula after 15 min of in vitro digestion. No clear patterns in terms of digestion status were observed.

After 15 min of in vitro digestion, the quantity of pIgR peptides was highest in iPF-P and for GlyCAM1 peptides it was highest in iPF-L.

Of the eHF samples, eHF-L showed higher quantities of both pIgR and GlyCAM1 peptides, while the opposite was found for the iPF samples.

#### 3.1.4. Osteopontin

**Figure 6:**
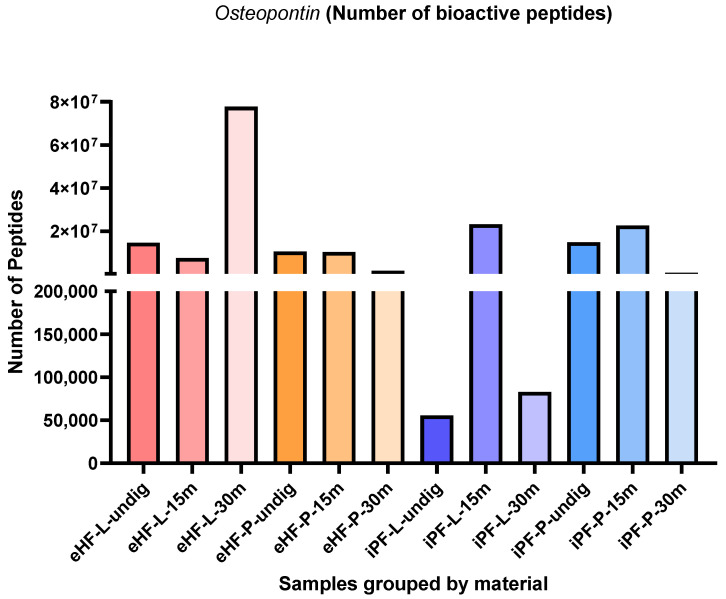
Number of osteopontin peptides per formula sample and digestion status.

The numbers of osteopontin peptides per sample and digestion status are presented in Figure 6. The highest quantity of osteopontin peptides was found in the eHF-L sample after 30 min of in vitro digestion (>77 × 10^6^ osteopontin peptides’ quant value), while the lowest quantities were found in undigested iPF-L (<56 × 10^3^ quant value) and iPF-L after 30 min of in vitro digestion (<83 × 10^3^ quant value).

In the iPF samples, the highest quantities of osteopontin peptides were found in samples after 15 min of in vitro digestion, while no clear pattern was observed for the eHF samples.

After 15 min of in vitro digestion, the quantities of osteopontin peptides were highest for both iPF-L and iPF-P (each >22 × 10^6^ quant value), whereas the quantities in the respective eHF samples were slightly lower (eHF-L: >7 × 10^6^, eHF-P: >10 × 10^6^ quant value).

Overall, the quantities of osteopontin peptides in the powder and liquid samples of both formula types were somewhat similar. In the eHF samples, eHF-L showed a higher quantity after 30 min of in vitro digestion. In the iPF samples, iPF-L showed a higher quantity after 15 min of in vitro digestion, whereas iPF-P was higher in quantity in the undigested state and after 30 min of in vitro digestion.

### 3.2. Antimicrobial Peptides

**Figure 7:**
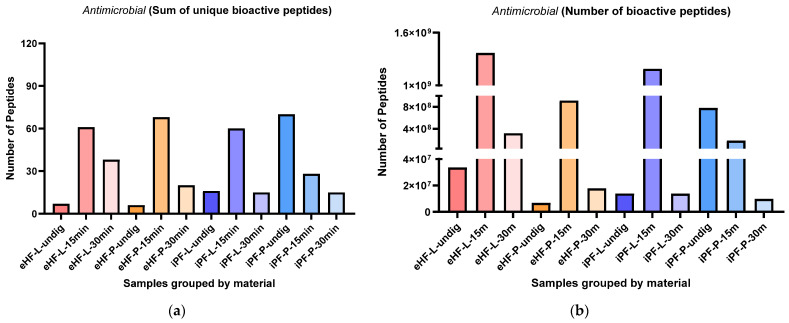
(**a**) Sum of unique bioactive peptides found among antimicrobial peptides per formula sample and digestion status. (**b**) Number of bioactive peptides found among antimicrobial peptides per formula sample and digestion status.

The sums of unique antimicrobial peptides per sample and digestion status are presented in Figure 7a. The highest sum of unique antimicrobial peptides was found in the undigested iPF-P sample (70 unique antimicrobial peptides), followed by eHF-P (68 peptides), eHF-L (61 peptides), and iPF-L (60 peptides), all after 15 min of in vitro digestion.

Overall, the highest sums of unique antimicrobial peptides were found after 15 min of in vitro digestion, except for the iPF-P sample, which had the highest number of unique peptides in the undigested state.

In the eHFs, the lowest numbers of unique antimicrobial peptides were found in the undigested states. In the iPFs, the lowest sums were found after 30 min of in vitro digestion; however, for iPF-L the number of peptides was similar to that in the undigested state.

Overall, there were no pronounced differences between the powder and liquid samples for both formula types (eHFs and iPFs).

The numbers of antimicrobial peptides per sample are presented in Figure 7b. The highest quantity of antimicrobial peptides was found in eHF-L after 15 min of in vitro digestion (>13 × 10^6^ antimicrobial peptides’ quant value), followed by iPF-L (>11 × 10^6^ peptides’ quant value), and eHF-P (>913 × 10^6^ quant value), both after 15 min of in vitro digestion, and undigested iPF-P (>780 × 10^6^ quant value).

Across all samples, the highest quantities of antimicrobial peptides were found after 15 min of in vitro digestion; except for iPF-P, where the highest amount was found in the undigested state.

In the eHF samples, the lowest quantities of antimicrobial peptides per sample were found in the undigested state. In the iPFs, the lowest quantities were found after 30 min of in vitro digestion, but for iPF-L the amount was similar to that in the undigested state.

Overall, there were no distinct differences between the powder and liquid samples of both formula types (eHFs and iPFs).

### 3.3. Antioxidative Peptides

**Figure 8:**
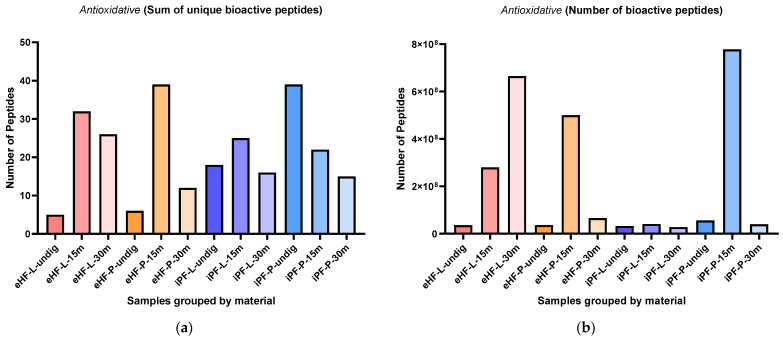
(**a**) Sum of unique bioactive peptides found among antioxidative peptides per formula sample and digestion status. (**b**) Number of bioactive peptides found among antioxidative peptides per formula sample and digestion status.

The sums of unique antioxidative peptides per sample and digestion status are presented in Figure 8a. The highest sum of unique antioxidative peptides was found in eHF-P after 15 min of in vitro digestion and in undigested iPF-P (each with 39 unique antioxidative peptides), followed by eHF-L after 15 min of in vitro digestion (32 peptides), eHF-L after 30 min of in vitro digestion (26 peptides), and iPF-L (25 peptides) and iPF-P (22 peptides), both after 15 min of in vitro digestion.

Overall, the highest sums of unique antioxidative peptides were found after 15 min of in vitro digestion, except for the iPF-P sample, where the undigested state had the highest number of unique peptides.

In the eHFs, the lowest sums of unique antioxidative peptides were found in the undigested state. In the iPFs, the lowest sums were found after 30 min of in vitro digestion, but for iPF-L the sum was similar to the undigested state.

Overall, there were no pronounced differences between the powder and liquid samples for both formula types (eHFs and iPFs).

The numbers of antioxidative peptides per sample and digestion status are presented in Figure 8b. The highest quantity of antioxidative peptides was found in the iPF-P sample after 15 min of in vitro digestion (>1.3 × 10^9^ antioxidative peptides’ quant value), followed by eHF-L after 30 min of in vitro digestion (>665 × 10^6^ quant value), and eHF-P (>499 × 10^6^ quant value) and eHF-L (>280 × 10^6^ quant value), both after 15 min of in vitro digestion.

Across all samples, the highest quantities of antioxidative peptides were found after 15 min of in vitro digestion; except for eHF-L, where the highest amount was found after 30 min of in vitro digestion.

In the eHFs, the lowest quantities of antioxidative peptides were found in the undigested state, and in the iPFs after 30 min of in vitro digestion.

Overall, there were no distinct differences between the powder and liquid samples for both formula types (eHFs and iPFs) except for eHF samples after 30 min of in vitro digestion, where eHF-L showed a higher quantity of peptides and for iPF samples after 15 min of in vitro digestion, where iPF-P showed a higher quantity of peptides.

Additionally, a high amount of the peptide TQTPVVVPPFLQPE (amino acids 93–106 of β-casein) was found in eHF-L after 30 min of in vitro digestion (>585 × 10^6^ for quant value) and in iPF-P after 15 min of in vitro digestion (>746 × 10^6^ as quant value).

### 3.4. Wound Healing Peptides

**Figure 9:**
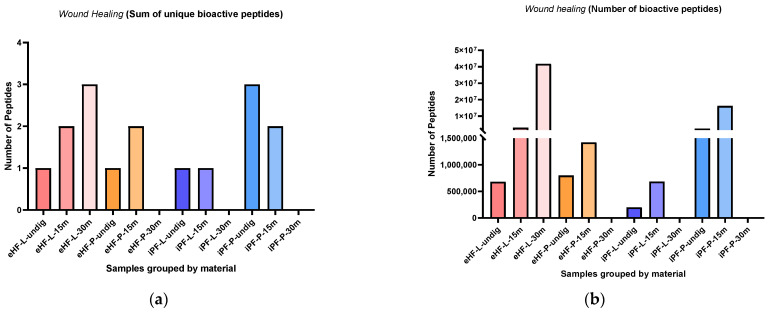
(**a**) Sum of unique bioactive peptides found among wound healing peptides per formula sample and digestion status. (**b**) Number of bioactive peptides found among wound healing peptides per formula sample and digestion status.

The sums of unique wound healing peptides per sample and digestion status are presented in Figure 9a. The highest sum of unique wound healing peptides was found in eHF-L after 30 min of in vitro digestion and in undigested iPF-P (each with three unique wound healing peptides). No wound healing peptides were found in the samples of eHF-P, iPF-L, and iPF-P, all after 30 min of in vitro digestion.

Overall, the sum of unique wound healing peptides in the powder and liquid samples of both formula types was somewhat similar. In the eHF samples, eHF-L showed a higher quantity after 30 min of in vitro digestion. In the iPF samples, iPF-P showed a higher quantity in the undigested state and after 15 min of in vitro digestion.

The numbers of wound healing peptides per sample and digestion status are presented in Figure 9b. The highest quantity of wound healing peptides was found in eHF-L after 30 min of in vitro digestion (>41 × 10^6^ wound healing peptides’ total quant value), followed by iPF-P after 15 min of in vitro digestion (>16 × 10^6^ quant value).

Across all samples, the highest quantities of wound healing peptides were found after 15 min of in vitro digestion; except for eHF-L, where the highest amount was found after 30 min of in vitro digestion.

In the eHF samples, the liquid form showed a higher quantity of wound healing peptides than the powder form, except for the undigested samples which were similar in quantity. In the iPF samples the powder form showed a higher quantity of peptides.

### 3.5. Cholesterol Metabolism Regulation Peptides

**Figure 10:**
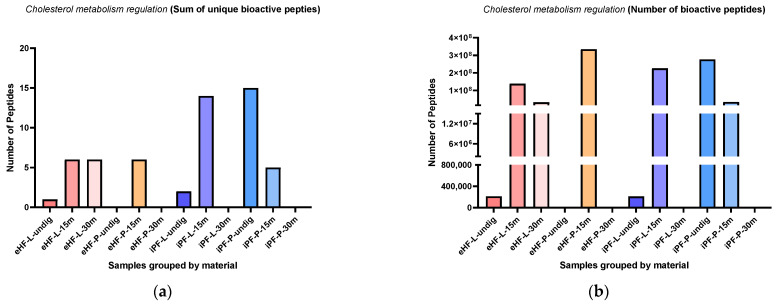
(**a**) Sum of unique bioactive peptides found among cholesterol metabolism regulation peptides per formula sample and digestion status. (**b**) Number of bioactive peptides found among cholesterol metabolism regulation peptides per formula sample and digestion status.

The sums of unique cholesterol metabolism regulation (CMR) peptides per sample and digestion status are presented in Figure 10a. The highest sum of unique CMR peptides was found in the undigested iPF-P sample (15 unique CMR peptides), followed by iPF-L after 15 min of in vitro digestion (14 peptides). No CMR peptides were found in samples of undigested eHF-P, and eHF-P and iPF-P after 30 min of in vitro digestion. All other samples had a maximum sum of six unique peptides.

Across all samples, the highest sums of unique CMR peptides were found after 15 min of in vitro digestion; except for iPF-P, where the highest sum was in the undigested state.

The numbers of CMR peptides per sample and digestion status are presented in Figure 10b. The highest quantities of CMR peptides were found in eHF-P after 15 min of in vitro digestion (>334 × 10^6^ cholesterol regulation peptides’ quant value), followed by undigested iPF-P (>276 × 10^6^ peptides), and iPF-L after 15 min of in vitro digestion (>225 × 10^6^ as quant value). No CMR peptides were found in the undigested eHF-P sample, nor in eHF-P, iPF-L, and iPF-P, all after 30 min of in vitro digestion.

Overall, there were no marked differences between the powder and liquid samples for both formula types (eHFs and iPFs).

### 3.6. Peptides Affecting Salmonella Antivirulence Activity

**Figure 11:**
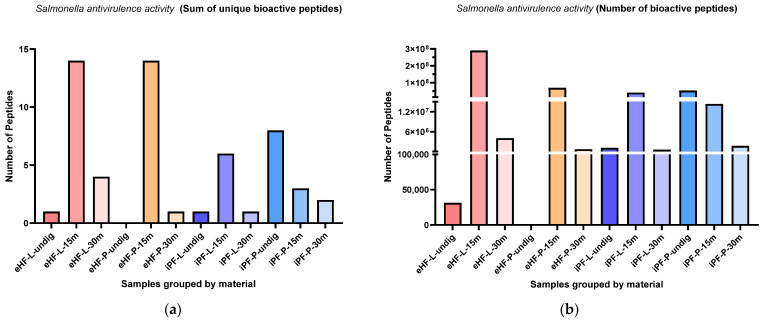
(**a**) Sum of unique bioactive peptides found among *Salmonella* antivirulence activity peptides per formula sample and digestion status. (**b**) Number of bioactive peptides found among *Salmonella* antivirulence activity peptides per formula sample and digestion status.

The sums of unique *Salmonella* antivirulence activity (SAA) peptides per sample and digestion status are presented in Figure 11a. The highest sums of unique SAA peptides were found in eHF-L and eHF-P (14 unique SAA peptides each), both after 15 min of in vitro digestion. No SAA peptides were found in undigested eHF-P. All other samples had a maximum sum of under eight unique peptides.

Across all samples, the highest sums of unique SAA peptides were found after 15 min of in vitro digestion; except for iPF-P, where the highest sum was in the undigested state.

The numbers of SAA peptides per sample and digestion status are presented in Figure 11b. The highest quantities of SAA peptides were found in eHF-L after 15 min of in vitro digestion (>289 × 10^6^ SAA peptides’ quant value). All other samples with SAA peptides had quantities below 71 × 10^6^.

Across all samples, the highest quantities of SAA peptides were found after 15 min of in vitro digestion; except for iPF-P, where the highest amount was found in the undigested state.

In the eHF samples, eHF-L showed a higher quantity of SAA peptides across all digestion states. In the iPF samples, there were no distinct differences between the powder and liquid forms.

### 3.7. Peptides Known from Colostrum

**Figure 12:**
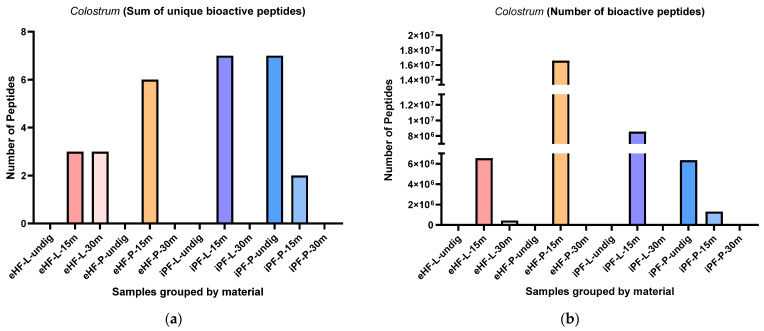
(**a**) Sum of unique bioactive peptides found among peptides known from colostrum per formula sample and digestion status. (**b**) Number of bioactive peptides found among peptides known from colostrum per formula sample and digestion status.

The sums of unique peptides known from colostrum per sample and digestion status are presented in Figure 12a. The highest sums of unique peptides known from colostrum were found in undigested iPF-P and iPF-L after 15 min of in vitro digestion (each with eight unique peptides known from colostrum), followed by eHF-P after 15 min of in vitro digestion (six peptides). No peptides known from colostrum were found in undigested eHF-L, eHF-P, and iPF-L, nor in eHF-P, iPF-L, and iPF-P, all after 30 min of in vitro digestion. All other samples had a maximum sum of three unique peptides.

Overall, the highest sums of unique peptides known from colostrum were found in samples after 15 min of in vitro digestion; except for the iPF-P, which had the highest number of unique peptides in the undigested state.

Overall, there were no marked differences between the powder and liquid samples for both formula types (eHFs) and (iPFs); except for eHF and iPF samples after 15 min of in vitro digestion, where eHF-P and iPF-L showed a higher variety of peptides.

The numbers of peptides known from colostrum per sample and digestion status are presented in Figure 12b. The highest quantity of peptides known from colostrum was found in eHF-P after 15 min of in vitro digestion (>16 × 10^6^ peptides known from colostrum quant value), followed by iPF-L after 15 min of in vitro digestion (>8 × 10^6^ quant value).

Across all samples, the highest quantities of peptides known from colostrum were found after 15 min of in vitro digestion; except for iPF-P, where the highest amount was found in the undigested state.

Overall, there were no pronounced differences between the powder and liquid forms for both formula types (eHFs and iPFs).

## 4. Discussion

Bioactive peptides result from the digestion of proteins in human milk and play an important role in the maintenance of gut health in infants. Formula-fed infants also receive—and benefit from—bioactive peptides, which stem from bovine milk proteins [28]. Their quantity and quality are impacted by the degree of processing the IF has undergone [29].

In this study, two types of IF, eHF and iPF (in both liquid and powder forms), were subjected to in vitro gastrointestinal digestion and analyses of bioactive peptides were performed at the undigested stage and after 15 and 30 min of in vitro digestion (mimicking the gut of a young infant and of an older infant). Overall, the form of IF (liquid vs. powder) had little effect on the number of bioactive peptides, but the effect of the type of IF (extensively hydrolyzed vs. intact protein) was more decisive. While in a previous study only very few bioactive peptides were found in undigested and digested extensively hydrolyzed formula [8], in this study, a variety of peptides with potential bioactive properties were found in all samples. This suggests that different properties of the hydrolyzed proteins are retained depending on the production process of the hydrolysate and IF. Across all samples in this study immuno-regulatory peptides, followed by antimicrobial and antioxidative peptides, were the most frequently found, as were peptides known for their roles in promoting wound healing, increasing mucin secretion, regulating cholesterol metabolism, and preventing bacterial infection. Peptides typically found in yoghurt and colostrum were also identified in some formula samples. Similar results were observed in other studies for antimicrobial [30] and antioxidative peptides [31], and peptides known from colostrum [32].

The most common peptide type across all samples was casein peptides, especially β-casein. Other types found were from whey proteins. Since all whey protein concentrates are either produced by acidification or the result of industrial cheese processes, they typically also contain small quantities of casein proteins [33,34], which correlates with the results in this study. Casein peptides will also be present in whey protein concentrates before hydrolysis (derived partly from enzymatic activity in the udder and partly from rennet activity during cheese production) [35,36]. Further, this is similar to what has been found for whey proteins and casein in human milk [4,5]. Notably, as bioactive peptides are broken down into protein fragments, they do not lead to allergic sensitization; on the contrary, they can act as immuno-modulators [37].

The variety of immuno-regulatory peptides was comparable in eHF and iPF samples; however, the quantity of immuno-regulatory, anti-inflammatory and osteopontin peptides was higher in the eHF samples. Osteopontin is a natural component of human milk [38], acts as a Th1 cytokine, and is known to have a role in setting up a tolerogenic milieu [39]. Furthermore, Lactoferrin is one of the major human milk proteins [4,40]. It is an ion-binding glycoprotein enhancing intestinal iron absorption, promotes gut health, and plays a crucial role in modulating the development of infants’ immune systems [41,42]. Lactoferrin peptides were found in almost all the samples analysed. Interestingly, Lactoferrin peptides were not found in all stages of digestion in eHF-P samples. Lactoferrin peptides were not found at 15 min of in vitro digestion but were found in undigested samples and after 30 min of in vitro digestion. This may be attributed to the release or degradation of varying bioactive peptides at different stages during the digestive process. The quantities of peptides with functions related to T cell response were also comparable in eHF and iPF samples, whereas the quantity of pro-inflammatory peptides was lower in eHF samples. Pro-inflammatory peptides in eHF samples were only found after 30 min of in vitro digestion (for liquid and powder). These findings suggest that eHFs, in both liquid and powder form, are more likely to trigger an anti-inflammatory response than iPFs.

The variety and quantity of antimicrobial peptides was comparable in eHF and iPF samples, with the overall highest amounts being found in three samples after 15 min of in vitro digestion (eHF-L, eHF-P, and iPF-L) and in the undigested iPF-P sample. It is therefore assumed that the 15 min digestion time of IF samples exposed their ‘encrypted’ bioactive peptides [43,44], which were detected in eHF-L, eHF-P, and iPF-L. While a longer digestion time (30 min) can lead to such strong cleavage that no more peptides are detectable. Further studies are required to clarify why the peptide digestion pattern of iPF-P differs in this regard.

The variety of antioxidative peptides was comparable in eHF and iPF samples; however, the overall quantity was higher across the eHF samples, although the highest quantity was found in an iPF-P sample after 15 min of in vitro digestion. A high amount of the peptide TQTPVVVPPFLQPE (amino acids 93–106 of β-casein) was found in two samples: eHF-L after 30 min and iPF-P after 15 min of in vitro digestion. This bioactive peptide is linked to several functions: antioxidative, anti-hypertensive, or ACE-inhibitory, which consequently can aid in lowering blood pressure [22].

The varieties of wound healing peptides were somewhat comparable in eHF and iPF samples; however, more iPF samples showed no traces of wound healing peptides (iPF-L and iPF-P, both after 30 min of in vitro digestion) than eHF samples (only eHF-P after 30 min of in vitro digestion). The overall quantities were, however, higher in the eHF samples.

Peptide assays for wound healing can be carried out using fibroblast cells in culture [45]. Fibroblasts are known to be involved in chronic gut inflammation, and thus, the ability to improve wound healing in the gut with bioactive peptides may contribute to gut homeostasis and gut health [46]. Intestinal injury accompanied by impaired mucosal wound healing as it is seen, e.g., in preterm infants with necrotizing enterocolitis (NEC) [47], or in patients with chronic inflammatory bowel disease (IBD) [48,49], or Crohn’s disease (CD) [49,50], may also be attenuated by such wound healing bioactive peptides. However, this hypothesis has yet to be confirmed.

The variety of peptides regulating cholesterol metabolism was higher in the iPF samples than the eHFs; however, no peptides were found in two samples each (undigested eHF-P after 30 min of in vitro digestion and iPF-L and iPF-P, both after 30 min of in vitro digestion). Interestingly, the overall quantities of cholesterol metabolism regulation peptides were comparable in the eHF and iPF samples and highest in the eHF-P sample after 15 min of in vitro digestion. This result is in line with findings from the ongoing PADI study [51]. In this study, the blood cholesterol levels in infants fed with IFs similar to eHF-P and iPF-P at one year of age were compared. Infants fed eHF-P had significantly lower blood cholesterol levels than infants fed iPF-P (*p* < 0.05) [51] (unpublished data), which was associated with a higher quantity of bioactive peptides for cholesterol metabolism regulation in eHF-P compared with iPF-P after in vitro infant digestion.

The variety of *Salmonella* antivirulence activity (SAA) peptides was higher in the eHF samples than in the iPF samples; however, no peptides were found in the undigested eHF-P sample. Interestingly, the overall quantities of SAA peptides were comparable in the eHF and iPF samples, with the highest quantity found in the eHF-L sample after 15 min of in vitro digestion. *Salmonella* infection (salmonellosis) is a common bacterial disease caused by *Salmonella typhimurium* and characterized by inflammation of the intestinal tract [52,53]. Consequently, the higher levels of SAA peptides in the eHF samples could benefit infants fed eHF, resulting in a lower susceptibility to *Salmonella* infections and outbreaks.

Peptides known from bovine colostrum were found in three of the six IF samples, for each of the eHFs and iPFs. The variety of peptides known from colostrum was higher in the iPF samples than the eHFs, but the maximum number of unique peptides in the iPFs was seven compared with the maximum of six unique peptides in the eHFs. In contrast, the overall quantities of peptides known from colostrum were higher in the eHF samples. Colostrum is the initial milk secreted by mammals during the first few days after the birth of their offspring and is a source of biologically active peptides with health-promoting effects [54].

An overall interesting result is the generally high amounts and varieties of bioactive peptides found in the undigested iPF-P samples. Across all the peptide types described above, undigested iPF-P had the highest variety for six of the described nine bioactivities and the highest quantity for two of the 15 quantity analyses, therefore often scoring higher than the respective digested samples. One possible explanation for this finding is the methodology used for sample preparation, whereby all samples were further subjected to trypsinization, thus creating smaller peptide sizes which were detectable in LC–MS analyses. The less a sample is processed from one container to another at room temperature, the fewer peptides will be lost during preparation. Protein degradation already begins above −80 °C; therefore, peptides resulting from this degradation are less likely to be identified by the software. This would be the case, even if the mass spectrometer detects them. Further, the resulting peptides can have such small sizes that as a consequence they might get washed away during the process.

This study had both strengths and limitations. A series of different IFs were analysed as biological duplicates, but no statistics were compiled, and the analysis was limited to a descriptive comparison of the peptides found in the different samples. Samples were analyzed as total formula, which is in contrast to analyzing the protein itself, and additionally included fat molecules. This could potentially have affected the extraction of proteins in the formulas, due to the fat moieties in the aqueous buffer used for the tryptic digestion, which consisted of 50 mM triethyl ammonium bicarbonate in H_2_O. As a consequence, the proteins in the formulas would have reacted with a heightened stickiness and thus would have been less detectable in the LC–MS analysis. Samples were analyzed by LC–MS, which provides a relatively unbiased global view of the IFs’ bioactive peptide composition. Further, this method can be used to analyze all samples in the same manner; thus, analytical bias towards the various formulations is limited. However, by using LC–MS, certain hydrophilic or small peptides (<approximately four amino acids) may not have been registered during the 10 kDa molecular weight separation. This may also have affected the tryptic peptides produced from the above 10 kDa peptide fraction. Consequently, the probability of the formula samples containing even higher numbers of bioactive peptides is plausible. Due to these limitations in using the LC–MS system, an analysis of potential biologically active peptides with other methods is recommended in future studies.

In summary, the overall highest quantities and varieties of bioactive peptides were found in the samples of eHF-L after 15 min of in vitro digestion, eHF-L after 30 min of in vitro digestion, iPF-L after 15 min of in vitro digestion, and undigested iPF-P. In contrast, the lowest overall quantities and varieties were found in three of the samples digested for 30 min (eHF-P, iPF-L, and iPF-P), as well as in the undigested eHF-P samples. Interestingly, in contrast to results from a previous study, eHF samples showed similar compositions of bioactive peptides overall as iPF samples, with a tendency to higher variety and quantity across peptide types. The high amounts of immuno-regulatory, antimicrobial, and antioxidative peptides across most samples further indicate possible contributions to protection against infection, to healthy gut microbiomes, and to immunological development in infants.

## 5. Conclusions

In this study two types of IF, eHF and iPF (in both liquid and powder forms), were subjected to in vitro gastrointestinal digestion, and analyses of bioactive peptides were performed. In all samples, varieties of peptides with potential bioactive properties were found. Immuno-regulatory peptides, followed by antimicrobial and antioxidative peptides, were the most frequently found, as were peptides known for their roles in promoting wound healing, increasing mucin secretion, regulating cholesterol metabolism, and preventing bacterial infection. Peptides typically found in yoghurt and colostrum were also identified in some formula samples. The overall highest quantities and varieties of bioactive peptides were found in two eHF and in two iPF samples.

## Figures and Tables

**Table 1:** Description of investigated materials.

No.	Abbreviation	Description	Pre/Probiotics
1	eHF-L	HiPP HA infant formula (HiPP Pre HA COMBIOTIK^®^, liquid)	GOS
2	eHF-P	HiPP HA infant formula (HiPP Pre HA COMBIOTIK^®^, powder)	GOS, *L. ferm.* CECT 5716
3	iPF-L	HiPP standard cow’s milk-based infant formula (HiPP Pre Bio COMBIOTIK^®^, liquid)	GOS
4	iPF-P	HiPP standard cow’s milk-based infant formula (HiPP Pre Bio COMBIOTIK^®^, powder)	GOS, *L. ferm.* CECT 5716

HA infant formula based on extensively hydrolyzed cow’s milk (whey) protein (Peptigen^®^ IF-3080, Arla Foods Ingredients (Videbæk, Denmark)). Standard infant formula based on cow’s milk intact protein. Infant formulas were provided by HiPP GmbH & Co. Vertrieb KG (Pfaffenhofen, Germany). eHF—extensively hydrolyzed formula; iPF—intact protein formula; L—liquid; P—powder; GOS—Galactooligosaccharides; *L. ferm.* CECT 5716—*Limosilactobacillus fermentum* CECT 5716 (originally obtained from human milk).

**Table 2:** Most common (top 25%) bioactive peptides identified in samples of IFs after 15 min of in vitro digestion that showed sequence homology with a known bioactive milk peptide from the BIOPEP-UWM database.

Parental Protein	Residue	Peptide	eHF-L	eHF-P	iPF-L	iPF-P	Function
Beta-casein	144–154	DVENLHLPLPL			Y	Y	Antimicrobial
Beta-casein	123–128	EMPFPK			Y		Antimicrobial
Beta-casein	109–121	GVSKVKEAMAPKH	Y	Y			Antimicrobial
Beta-casein	121–128	HKEMPFPK			Y	Y	Antimicrobial
Beta-lactoglobulin	100–107	IDALNENK		Y	Y		Antimicrobial
Beta-lactoglobulin	19–26	IIVTQTMK		Y			Antimicrobial
Alpha-S1-casein	21–30	IKHQGLPQEV	Y	Y	Y		Antimicrobial
Alpha-S2-casein	94–103	QKALNEINQF	Y	Y			Antimicrobial
Beta-casein	32–42	SSSEESITRIN	Y				Antimicrobial
Alpha-S2-casein	163–176	TKKTKLTEEEKNRL	Y	Y			Antimicrobial
Beta-lactoglobulin	141–151	TPEVDDEALEK			Y	Y	Antimicrobial
Beta-casein	113–120	VKEAMAPK	Y				Antimicrobial
Alpha-S1-casein	30–37	VLNENLLR	Y	Y			Antimicrobial
Beta-casein	185–191	VLPVPQK				Y	Antimicrobial
Beta-lactoglobulin	108–116	VLVLDTDYK			Y	Y	Antimicrobial
Beta-casein	193–206	VPYPQRDMPIQAFL	Y	Y			Antimicrobial
Kappa-casein	183–190	VQVTSTAV	Y	Y			Antimicrobial
Beta-casein	208–224	YQEPVLGPVRGPFPIIV	Y	Y	Y	Y	Antimicrobial (bovine colostrum)
Beta-casein	144–154	DVENLHLPLPL			Y	Y	Antimicrobial (Salmonella antivirulence activity)
Beta-casein	109–121	GVSKVKEAMAPKH	Y	Y			Antimicrobial (Salmonella antivirulence activity)
Beta-casein	32–42	SSSEESITRIN	Y	Y			Antimicrobial (Salmonella antivirulence activity)
Alpha-S2-casein	189–196	FALPQYLK				Y	Antioxidant
Beta-casein	126–134	FPKYPVEPF			Y		Antioxidant
Alpha-S2-casein	95–103	KALNEINQF		Y			Antioxidant
Beta-casein	184–191	KVLPVPQK	Y			Y	Antioxidant
Alpha-S1-casein	42–50	PFPEVFGKE	Y	Y			Antioxidant
Beta-casein	157–169	SWMHQPHQPLPPT			Y		Antioxidant
Beta-casein	93–106	TQTPVVVPPFLQPE				Y	Antioxidant
Beta-casein	113–120	VKEAMAPK	Y		Y		Antioxidant
Alpha-S1-casein	159–164	YFYPEL					Antioxidant
Beta-casein	206–213	YQEPVLGP		Y			Antioxidant
Beta-casein	208–217	YQEPVLGPVR	Y	Y			Antioxidant
Alpha-S2-casein	105–111	YQKFPQY		Y			Antioxidant
Beta-lactoglobulin	25–30	GLDIQK			Y		Cholesterol regulation
Beta-casein	143–147	TDVEN	Y	Y	Y		Cholesterol regulation
Alpha-S1-casein	40–45	VAPFPE	Y	Y	Y	Y	Cholesterol regulation
Beta-casein	160–175	HQPHQPLPPTVMFPPQ	Y	Y			Immunomodulatory
Beta-casein	184–190	KVLPVPQ				Y	Immunomodulatory
Beta-casein	184–191	KVLPVPQK	Y	Y			Immunomodulatory
Alpha-S1-casein	157–164	LAYFYPEL			Y		Immunomodulatory
Beta-casein	207–224	LYQEPVLGPVRGPFPIIV			Y		Immunomodulatory
Alpha-S2-casein	122–126	NPWDQ			Y		Immunomodulatory
Beta-casein	209–213	QEPVL		Y			Immunomodulatory
Beta-casein	158–169	WMHQPHQPLPPT			Y		Immunomodulatory
Beta-casein	208–217	YQEPVLGPVR	Y	Y			Immunomodulatory
Beta-casein	208–224	YQEPVLGPVRGPFPIIV		Y			Immunomodulatory
Beta-casein	123–128	EMPFPK			Y		Increase mucin secretion
Alpha-S1-casein	159–164	YFYPEL					Increase mucin secretion
Beta-casein	75–81	YPFPGPI				Y	Increase mucin secretion
Beta-casein	185–191	VLPVPQK	Y	Y	Y	Y	Wound healing

BIOPEP-UWM—sequence database of Warmia and Mazury University; eHF—extensively hydrolyzed formula; iPF—intact protein formula; L—liquid; P—powder; Y—yes (presence of peptide in that sample).

## Data Availability

The data supporting the findings of this study will be provided by the corresponding author upon reasonable request.
